# Consumer Characterization of Wet- and Dry-Aged Mutton Flavor Profile Using Check-All-That-Apply

**DOI:** 10.3390/foods11203167

**Published:** 2022-10-11

**Authors:** Melindee Hastie, Damir Torrico, Zhenzhao Li, Minh Ha, Robyn Warner

**Affiliations:** 1 Faculty of Veterinary and Agricultural Sciences, The University of Melbourne, Parkville, VIC 3010, Australia; 2 Faculty of Agriculture and Life Sciences, Lincoln University, P.O. Box 85084, Lincoln 7647, New Zealand

**Keywords:** mutton, check-all-that-apply, lexicon, volatiles, fatty acid profile, consumer, sheep meat

## Abstract

The aim of this study was to assess if consumers could characterize wet- and dry-aged mutton flavor profiles using CATA (check-all-that-apply). A flavor lexicon was developed for mutton, and consumers assessed wet- and dry-aged mutton patties against this lexicon using CATA methodology. Results indicate that consumers most often associated caramel and roasted flavors with dry-aged patties, and “sheepy” and metallic flavors with wet-aged patties. Volatile analysis supported the consumer characterization as there were more Maillard reaction products, including pyrazines, which are associated with roasted and cooked flavors, found in the dry-aged patty volatile profile. More 1-octen-3-one, which is associated with metallic flavors, was found in the wet-aged patty volatile profile. These results provide validation that the lexicon utilized in this study (i) is suitable for the characterization of mutton flavor and (ii) will have applications for future investigations into the flavor components driving consumer liking for mutton.

## 1. Introduction

The Australian wool and sheep meat industry is seeking ways to add value to mutton (mutton in the context of this study refers to sheep meat from female or castrated male sheep older than 2 years). The Australian market only consumes approximately 10% of the mutton it produces, with the majority exported as a commodity product [1,2]. The eating quality of mutton is a challenge for processors/producers seeking to market mutton products, as it is less tender than lamb and has a stronger flavor, which can be objectionable to some consumers [3,4,5,6].

Holding sheep meat for a period post mortem in an anaerobic environment (wet-ageing) can improve its eating quality [7]. Wet-ageing is the most common commercial ageing method employed in Australia, which involves packaging whole primal cuts, such as loin or forequarter, under vacuum into plastic [8,9,10]. Once packaged, the meat is left in chillers and allowed to age for 5 to 10 days and during this time proteolysis commences, the meat begins to tenderize and favorable flavor changes occur due to the production of free amino acids and other meat flavor compounds and their precursors [11,12,13]. Under the anaerobic and acidic conditions characteristic of wet-ageing, most bacteria cannot grow, and LAB (lactobacillus sp.) proliferate, outcompeting potential pathogenic bacteria and making the wet-aged product safe to consume; however, LAB can cause a sour flavor in wet-aged meat [14,15]. Dry-ageing, on the other hand, is typically used for the production of premium niche beef products and is a potential novel application for sheep meat [16,17]. Dry-ageing involves hanging unpackaged primals or cuts in temperature and humidity controlled cabinets, usually with auxiliary fans to provide air movement within the cabinet. Ageing periods are often longer for dry-aged products (>21 days) [18,19]. Proteolysis also occurs during dry-ageing, but tenderization rates and flavor development can differ when compared to the wet-aged equivalent [20,21,22]. Dry-ageing is associated with increased positive flavor notes such as roasted, beefy, buttery flavors [19,20,22]. Several processes may be contributing to these favorable flavor changes; for example, moisture is lost from the meat during dry-ageing and, therefore, flavor compounds are more concentrated at the end of ageing [12], pH differences between wet- and dry-aged meat may affect flavor profiles [21,23] and the microflora associated with dry-aged meat (yeasts and molds) may contribute to flavor compounds and increase the rate of proteolysis [24,25].

Dry-ageing has been proposed as an intervention that may add value to mutton by increasing consumer liking for mutton. Recent investigations into the consumer response to wet- and dry-aged mutton have found that dry-ageing can indeed increase mutton liking for some consumers, but it can also reduce mutton liking for other consumers [26]. Hastie, Torrico, Hepworth, Jacob, Ha, Polkinghorne and Warner [26] also found that flavor is the most important driver of consumer liking for mutton followed by tenderness and juiciness; however, the consumer perceived flavor profile of wet- and dry-aged mutton has not been described. The development of a consumer-centric lexicon describing mutton flavor would support future investigation into the flavor component driving liking (or disliking) for mutton.

Recently, check-all-that-apply (CATA) methodologies have successfully been used with consumers to characterize red meat product flavor profiles [27,28]. It is proposed that this methodology may also capture the differences between dry- and wet-aged mutton flavor. Characterization of wet- and dry-aged mutton flavor profiles will enable articulation of the flavor benefits of dry-ageing mutton for branding and marketing purposes, and the future linking of flavor components to consumer liking or disliking of mutton products.

The aim of this study was to assess if consumers could characterize wet- and dry-aged mutton flavor profiles using CATA. Volatile and fatty acid analyses of the same wet- and dry-aged mutton samples was also conducted for comparison with the CATA results.

## 2. Materials and Methods

### 2.1. Consumer Sensory Testing

Approval was granted for this study by the University of Melbourne’s Human Research ethics committee, reference HREC1646413.4. All subjects gave their informed consent for inclusion before they participated in the study.

#### 2.1.1. Consumer Questionnaire Design

A 2-page sensory assessment form was used for the assessment of each sample. Two open-ended top-of-mind questions were given on the first page with space provided after each question for free text responses. Question 1 was: “in terms of odor, what is top-of-mind when you first smell the sample?”, while question 2 read: “in terms of flavor, what is top-of-mind when you first taste the sample?”. These questions served a dual purpose; firstly, to provide an indicator of the most intense odor and flavor characteristics the consumers were experiencing, and secondly, to capture any descriptor terms that consumers used but were not included in the CATA terms. The second page contained 16 CATA terms arranged in 2 columns with checkboxes using the heading “Please check all descriptors that apply to the sample”. The order of the terms was randomized for each individual consumer using the Research Randomiser application version 4.0 [29].

The CATA terms considered for inclusion in this study were initially developed from a review of the published literature characterizing the flavor and aroma profile of sheep meat; these terms were then validated through informal tasting sessions that were conducted at the University of Melbourne using untrained consumers. The final 16 terms were selected on the basis that they were descriptors the untrained consumer could understand easily and/or were terms used by the in-house tasters when describing the differences between wet- and dry-aged mutton samples. The final terms used, and their origins, are detailed in Table 1.

#### 2.1.2. Sample Preparation for Consumer Sensory Assessment

It is understood the sensory quality of sheep meat is influenced by factors such as animal age, pH, and production system [30,31,32,33,36,39]. Previous investigations comparing the consumer sensory response to wet- and dry-ageing of mutton found sensory differences due to ageing method (wet vs. dry) are relatively small compared to the effect of animal and production factors [43]. Therefore, the investigators elected to use a single carcass to fabricate the samples used in this study, so as to focus on differences due to the ageing method treatments and avoid confounding effects from animal and production factors.

A mutton carcass (female animal >2 years old) was sourced from a commercial meat wholesale outlet in Melbourne, Australia, at two days post mortem; before the carcass was prepared for delivery to the University of Melbourne, the carcass had been hung in the wholesalers chiller kept at 0–2 °C. For delivery to the University of Melbourne, the carcass was prepared by the wholesaler as a 6-way cut (2 × bone-in loins, 2 × bone-in forequarters and 2 × bone-in chump on legs) as described by the HAM (Handbook of Australian Meat) reference 4620 [44]. The forequarters (HAM ref. 4972) and the legs (HAM ref. 4800) were removed from the loins (HAM ref. 4860).

The left leg and forequarter were assigned to the dry-ageing treatment and placed on open steel mesh shelves in a Dry Ager DX1000 cabinet (Viking Food solutions, Epping Victoria, Australia) where ageing conditions were 80–85% RH and 0.0–2.5 °C for 19 days. The right-side forequarter and leg were assigned to the wet-ageing treatment; waxed polypropylene perforated cloth bone guard was applied to any sharp edges. They were then packaged under vacuum into Cryovac^®^ ultra-high abuse barrier bags (film thickness = 100 µm, size = 350 mm × 600 mm, oxygen transmission rate of 7 cm^3^ O_2_/m^2^/day @23 °C and 0% R.H., vapor transmission rate of 3 g/m^2^/day @38 °C) and aged for 19 days. The wet-ageing chiller temperature ranged from 0.0–2.0 °C. At the end of ageing, the wet- and dry-aged primals were trimmed (that is, any glands or imperfections were removed from the primal) and then the primals were deboned; the leg was deboned as described for HAM ref. 5060 and the forequarter deboned as described for HAM ref. 5047 [44].

In order to reduce any texture variation in the mutton samples, to ensure a focus on flavor and to provide homogenous samples for sensory testing, each treatment (wet-aged and dry-aged) involved mincing the deboned forequarter and leg using a Kenwood Meat Grinder MG450 type MG47 (Target, NSW, Australia); the meat was initially run through the mincer using the largest screen (8 mm) then mixed by hand on a stainless steel benchtop and the mixed coarse mince was then re-run through the mincer using the medium screen (4.5 mm) and mixed again on the stainless steel benchtop. Then, 200 g portions of the mince were weighed and formed into patties using an FED patty press mold (APEX Co. Pty. Ltd., Melbourne, Australia), each patty having a circumference of 100 mm and a height of 25 mm. The formed burgers were placed onto serving trays, covered in plastic film and kept chilled until sensory testing the following day; there were 22 patties prepared for each ageing method treatment. Two patties for each treatment and 500 g of raw mince from each treatment were retained for chemical analysis (pH, moisture, fatty acid profile and volatile analysis). These subsamples were vacuum-packed and stored at −70 °C until analysis.

#### 2.1.3. Conduct of Consumer Sensory Sessions

Sequential consumer sessions, with four participants in each session and each session of 15 min duration, were run during one day over a 6-h period. Consumers (*n* = 72) either nominated themselves after reading signage advertising the tasting session or they chose to participate after being approached by members of the research team. Nominees were excluded if they indicated they did not eat sheep meat or they had any food allergies or intolerances. All participants were provided a printed plain language statement outlining the study objectives and contact personnel; they were also verbally briefed on the study objectives, protocols for managing participant confidentiality and the anticipated time commitment. Upon consenting to participate in a tasting and signing consent forms, participants were seated at a table in groups of four where they completed a demographic survey, as described in Hwang et al. [45]. Before tasting commenced, they were briefed on the tasting procedure, how to complete the top-of-mind and CATA questionnaire and how to cleanse their palette before assessing each sample. Water, dry crackers, white plastic disposable cutlery and paper napkins were provided for each participant along with the CATA questionnaire and a pen. Once sample cooking commenced, they were instructed not to engage in conversation with fellow participants during the tasting. Each consumer tasted 2 samples (1 × wet-aged and 1 × dry-aged) with the presentation order of the two samples alternated for each session. The samples were served to all 4 participants simultaneously and they were prompted to lift the foil covering the sample, smell the sample and respond to question 1, and then to taste the sample, respond to question 2 and then complete the CATA for assessment for that sample.

#### 2.1.4. Cooking Method

The Meat Standards Australia grill cook method was utilized in this study [7]. Samples were cooked fresh for each sensory session. Cooking of samples commenced once 4 participants were seated for a sensory session. Patties were grilled on a preheated clamshell grill (Silex, Marrickville, Australia) with the top plate set to 185 °C and the bottom plate set to 195 °C; the top plate was closed 30 s after grilling commenced. Patties were cooked to an internal temperature of 65 °C. Once internal temperature of the patties reached 65 °C, the patties were removed from the grill and cut into quarters. Each portion was placed on a labelled plain white disposable plastic plate, covered with foil and then presented immediately to the consumers.

### 2.2. Chemical Analysis

#### 2.2.1. pH and Total Moisture

A frozen subsample (approx. 40 g) of raw wet- and dry-aged mince was thawed overnight at 6 °C in a sealed 50 mL falcon tube, and pH was determined in triplicate for each treatment using a spear-head pH probe (Ionode IJ44) attached to WP-80 pH-mV-temperature meter with attached temperature probe and automatic temperature compensation (TPS Pty Ltd., Brisbane, Queensland, Australia). Calibration was conducted using pH = 4.0 and 7.0 buffers.

Total moisture content (TM) of the raw and cooked mince (subsampled from the volatile analysis samples, see below) were determined in triplicate by oven drying. Approximately 4 g of the frozen fine mince was weighed into a foil dish and dried at 105 °C until a constant weight was reached, as described in Honikel [46]. TM % (total moisture) was calculated as described below:

TM (%) = ((Wt0 − Wt1)/Wt0) × 100(1)

Wt0 = the weight (g) of LL sample before drying,
Wt1 = the weight (g) of LL sample after drying.

#### 2.2.2. Fatty Acid Methyl Ester (FAME)

Muscle fatty acids contribute to the generation of several important odor active volatile compounds during the storage and cooking of meat [47], and the effect of dry-ageing on mutton fatty acids is unknown. Therefore, the FAME profile for the raw wet- and dry-aged patties was determined using the methodology outlined below.

For both treatments (wet- and dry-ageing), raw patty mince was analyzed for FAME using the methods described in Ponnampalam et al. [48] and Ponnampalam et al. [49]. All reagents and chromatography supplies were purchased from Sigma-Aldrich, Macquarie Park, New South Wales, Australia. Chemicals used in the analysis included internal standard (nonadecanoic acid methyl ester (C19:0) part no. 74208-1G), reagent-grade potassium hydroxide (part no. 1050331000), HPLC-grade methanol (part no. MA004-2.5L-J), AR-grade sulfuric acid (part no. 258105-2.5L-PC), reagent-grade hexane (RP1083-G2.5L), external standard fatty acid methyl ester (FAME) reference standard C8-C24 mix (part no. CRM18918).

Duplicate 1.0 g aliquots of frozen ground raw sample for each treatment (wet- and dry-aged) were weighed into 10 mL test tubes, 1 mL of internal standard (0.5 mg/mL nonadecanoic acid methyl ester in methanol) was added, followed by 0.7 mL of 10% KOH in water and 5.3 mL of methanol. Tubes were then mixed on a vortex mixer. The tubes were incubated at 55 °C for 1.5 h and shaken vigorously every 20 min. They were then cooled to room temperature under running water and then 0.6 mL of 24 N sulfuric acid in water was added and tube contents mixed. The incubation, shaking and cooling steps were repeated, then 3 mL of hexane was added to the tube and the contents were mixed for 5 min. Tubes were then centrifuged at 2000 rpm for 10 min with 1 mL of hexane layer collected into a 2 mL GC vial ready for analysis.

GC analysis was conducted on an Agilent 7890B GC system fitted with an Agilent 7693 autosampler, a flame ionization (FID) detector and a 25 m SGE-70 capillary column, internal diameter of 0.32 mm, 0.5 μm film thickness (part number 054606, Trajan Scientific and Medical, Ringwood, Australia). Helium was used as the carrier gas at a flow rate of 0.3 mL/min and the FID was supplied with 30 mL/min hydrogen, 300 mL/min air and nitrogen make-up gas at 30 mL/min. The GC was programmed for split injection (30:1) with the injector maintained at 260 °C throughout, and the FID at 260 °C. The oven program was initiated at 140 °C, where it was held for 5 min, then increased at 4 °C/min until it reached 240 °C, where it was held for 20 min. An injection volume of 1 μL was used for external standard and sample analysis.

Identification of fatty acids was based on retention time which was matched with the external FAME standard. Quantification of the sample FAMEs was conducted using FAME external standard curves over a concentration range 0.1 mg/g to 1.02 mg/g. All standard curves had an R^2^ value > 0.997; FAME results are expressed as FAME mg/g meat.

#### 2.2.3. Semi Quantitative Determination of Volatiles

Volatile analysis of freshly cooked wet- and dry-aged patties was based on the method of Gkarane et al. [50] with a slight modification: the SPME extraction was carried out at 60 °C instead of 90 °C to represent the temperature of freshy cooked meat at the point of consumption. All reagents and standards were purchased from Sigma-Aldrich, Macquarie Park, New South Wales, Australia, and all chromatography supplies were sourced from Agilent, Mulgrave, Victoria, Australia. Certified standards included C7–C30 saturated alkanes mix (1000 μg/mL each compound, part no. 484451), 1,2 dichlorobenzene (part no. 240664-100 mL), 1-pentanol (part no. 76929), 2-methylpyrazine (part no. M75608), hexanal (part no. 18109), isovaleraldehyde (part no. 61848), dimethyl trisulfide (part no. 79592), 1-penten-3-ol (part no. 01984), 4-methy-1-pentanol (part no. M66951), trans-2-heptenal (part no. 324140), isobutyraldehyde (part no. 240788), trans-2-octenal (part no. 52464), trans-2-nonenal (part no. 07592), octanal (part no. 52466), 1 octen-3-one (part no. 90963), 2-acetylpyrrole (part no. 247359), decanal (part no. D7384), nonanal (catalogue no. 442719), 4-methynonanoic acid (part no. W357405), p-cresol catalogue (part no. W233706), 4-ethyoctanoic acid (part no. W380008), 4-methyl octanoic acid (part no. W357502) and 3-methylindole (part no. 90961).

The patties retained for volatile analysis were defrosted overnight in the vacuum packaging at 6 °C and cooked the next morning using the cooking method described in Section 2.1.4. Upon reaching an internal temperature of 65 °C, the patty was removed from the hot plate and cut in half; one half was weighed into a 500 mL capacity Nutribullet cup and an equal weight of >99.0% anhydrous sodium sulphate (Sigma-Aldrich part number 239313) was immediately added to the cup. The cup was then sealed using the blade assembly, and the sample homogenized on a 900 W Nutribullet blender model NB9-0507 (Target, Brisbane, Queensland, Australia) for 30 s. The remaining cooked patty was retained for moisture determination, as described in Section 2.2.1. Immediately after blending, 4 replicate 4 g aliquots of the homogenate were weighed into 20 mL capacity headspace vials (Agilent part number 5188-2753) and sealed with PTFE-lined screw caps (Agilent part number 5188-2759). An amount of 2 µL of 6.15 ng/mL 1,2 dichlorobenzene internal standard was added to a 150 µL vial insert positioned in the headspace vial to ensure fiber integrity throughout the analytical run. An external 1,2 dichlorobenzene standard of the same concentration was also run.

Analysis of the sample headspace was conducted using an Agilent 5977B GC-MS in splitless mode, fitted with a PAL 3 RS1 120 autosampler (CTC instruments) and a HP-5MS 30 m × 0.25 µm film × 0.25 mm internal diameter column with a helium flow of 1 mL/min. The samples were equilibrated at 60 °C with gentle agitation for 45 min before extraction. The headspace was then extracted for 45 min with a 2 cm DVB/CAR/PDMS SPME fiber (Sigma-Aldrich part no. 57299-U). The fiber was desorbed in the injector for 8 min at 250 °C. The GC oven was held at 40 °C for 5 min and then ramped up at 4 °C/min until the oven reached 280 °C, where it was held for 5 min. Total ion chromatogram (TIC) data were collected over the mass-to-charge ratio range of 33 to 230 m/z. Tentative compound identification was via retention time matching with the purchased external standards or published Kovats retention indices (LRI); compound identity was confirmed by spectral matching using the NIST library (version 17.0) or with the certified standard. Quantification of identified compounds was based on the selected ion peak areas. An assumed response factor of 1 peak area unit = 1 ng of compound with results expressed as ng/g of meat.

### 2.3. Data Analysis

The completed surveys were reviewed for any erroneous or incomplete responses; hence, three consumers’ responses were removed from the analysis. For questions 1 and 2 of the sensory assessment (top-of-mind questions), all the participants’ responses were transcribed into Excel, and reviewed for any themes. Upon reviewing the responses for both the dry-aged and wet-aged samples, it was found that participants used a number of similar terms to describe a single component of the odor/flavor profile; therefore, groups were created to encompass these similar terms. For the unique taste and odor associated with “sheep meat”, the terms “lamb’, “lamby”, “mutton”, “sheep” and “sheepy” were grouped; similarly, the group formed for “cooked” included the terms “BBQ”, “roast”, “roasted” and “grilled”, the group “meat” included the terms “meat” and “meaty”, and the group “fat” included the terms “fat” and ‘fatty”. For dry- and wet-aged samples, the frequency of term usage was determined using the Wordcounter application (databasic.io; https://databasic.io/en/wordcounter/, accessed 19 October 2020) and the three most frequently used groups/terms were reported for the two treatments.

For the CATA analysis, selection frequency data were collated for each of the CATA terms for wet- and dry-aged patties. XLSTAT, 2021 version, (Addinsoft, New York, NY, USA) was used to compare the term frequencies for the two treatments using Cochran’s Q test for multiple pairwise comparisons.

Comparison of the pH, TM%, FAME and volatile data for the wet and dry patties was conducted using the two-sample *t*-test for independent samples, two-tailed test in XLstat with a significance level of 5%.

## 3. Results

### 3.1. Demographics

There were 38 male participants, 29 female participants and 2 participants who did not assign themselves a gender. All age categories were represented with 16 participants in the age range of 18–19 years, 4 participants between 20 and 25 years, 8 participants between 26 and 30 years, 6 participants between 31 and 39 years, 17 between 40 and 60 years, 13 between 60 and 70 years and 5 declined to report their age. Most participants described their cultural heritage as Australian (*n* = 33), with Asian the next largest group (*n* = 17), followed by European (*n* = 10), British (*n* = 7) and other (*n* = 2).

### 3.2. Responses to Top-of-Mind Questions (Q1 and 2 of the Sensory Assessment)

For wet- and dry-aged patties, frequency counts for the top 3 descriptor terms provided by participants in response to Q1 and 2 are presented in Table 2. For the wet-aged treatment odor descriptors, “sheep meat” terms were the most frequently selected, followed by the “meat” and “cooked” terms. For the dry-aged treatment odor descriptors, “sheep meat” was also selected the most frequently, followed by the “cooked” and then “meat” terms. In the taste descriptors for wet-aged, “sheep meat” and “fat” was most frequently selected, followed by the term “strong”, whereas for dry-aged it was found that “cooked” terms were the most frequently selected, followed by “fat” and “sheep meat”. Similar to the terms “sheep meat” and “fat”, the CATA includes the terms “sheepy” and fatty”. “Cooked” flavors would be similar to the CATA term “roasted”. The terms “meat” and “strong” were ambiguous in meaning and they did not have an equivalent CATA term.

### 3.3. Responses to “Top-of-Mind” Questions (Q1 and 2 of the Sensory Assessment)

The frequency of the CATA term selection for each of the treatments (wet and dry) are summarized in Table 3. Dry-aged patties were associated with increased caramel (*p* = 0.014) and roasted (*p* = 0.006) flavors, while wet-aged patties were associated with increased “sheepy” (*p* = 0.041) and metallic (*p* = 0.046) flavors and tended (*p* < 0.10) to be associated with increased barnyard (*p* = 0.058) and sour flavors (*p* = 0.059).

### 3.4. Chemical Analysis Results

#### 3.4.1. pH and Total Moisture Results

Table 4 summarizes the pH and moisture results. The pH of the dry-aged patties was 6.56, which was 0.1 pH unit higher than the wet-aged patties (pH = 6.46; *p* = 0.035). TM (%) of the raw wet-aged and dry-aged patties was 65.4% and 63.4%, respectively; however, the difference between the treatments was not significant (*p* = 0.167). TM (%) of the cooked wet- and dry-aged patties was 61.0 and 59.8%, respectively; the difference between the treatments was not significant (*p* = 0.477).

#### 3.4.2. Fatty Acid Methyl Esters (FAME) Results

The FAME results are shown in Table 5; methyl octanoate, methyl erucate and methyl lignocerate were not detected in either sample. There was no difference in the methyl docosanoate levels between the two treatments (*p* = 0.350). All other FAME values differed between the treatments (*p* < 0.05 for all). Methyl decanoate, methyl laurate, methyl tertradecanoate, methyl palmitate, methyl palmioleate, methyl linoleate, methyl arachidate and methyl linolenate were higher in the dry-aged patties compared to the wet-aged patties, whereas methyl octadeconate and cis-9-oleic acid methyl ester were higher in the wet-aged patties compared to the dry-aged.

#### 3.4.3. Volatile Analysis Results

Table 6 summarizes the effect of ageing method on the volatile concentration. For the aldehydes in general, the dry-aged treatment had higher concentrations of aldehydes than the wet-aged treatment (*p* < 0.05 for all), with the exception of 3-metylbutanal, (E)-2-octenal and tridecanal, where there was no difference in concentration between wet- and dry-aged treatment (*p* > 0.05 for all), and (E)-2-heptenal, which was higher in the wet-aged treatment compared to dry-aged (*p* = 0.001). For the detected alcohols, all but 2-Ethyl-1-hexanol were higher in the dry-aged treatment compared to the wet-aged treatment (*p* < 0.05 for all). The alkanes nonane, decane, unadecane, dodecane and tridecane were all found at higher concentration in the dry-aged treatment compared to wet-aged treatment (*p* < 0.05 for all). The aromatic hydrocarbons benzeneacetaldehyde and p-cresol were higher in the dry-aged treatment, while 1,4-dimethylbenzene (p-xylene) and acetophenone were higher in the wet-aged treatment (*p* < 0.05). Concentrations of 2-ethylfuran and 2-pentylfuran were higher in the dry-aged treatment (*p* < 0.05 for both). Ketone concentrations were generally higher in the dry-aged treatment (*p* < 0.001 for all), with the exception of 1-octen-3-one, which was higher in the wet-aged treatment (*p* = 0.003). For the indoles, only skatole (3-methylindole) was detected and then only in the wet-aged sample. For the organic acids, ethyl-butanoate was higher in the dry-aged treatment (*p* = 0.001), while hexanoic and octanoic acid were higher in the wet-aged treatment (*p* = 0.005 and 0.022, respectively). All pyrazines and 2-acetylpyrrole were higher in the dry-aged treatment compared to the wet-aged (*p* < 0.05 for all), with the exception of methyl-pyrazine. Dimethyl disulfide was higher in the wet-aged treatment (*p* = 0.001) and 2-acetyl-2-thiazoline was higher in the dry-aged treatment (*p* < 0.001). α-Terpineol was higher in the dry-aged sample (*p* = 0.011), while neophytadiene concentrations did not differ between treatments (*p* = 0.54).

## 4. Discussion

The CATA and top-of-mind results indicate that consumers can differentiate between wet- and dry-aged mutton patties and can characterize the different flavors and aromas associated with each ageing method. CATA assessments indicate that the dry-aged patty flavor was most associated with the positive flavor attributes of caramel and roasted flavor. Top-of-mind also indicated that dry-aged patties were mostly associated with a “cooked” flavor. The wet-aged patty CATA results indicate that “sheepy” and metallic flavors were most often selected, while the top-of-mind analysis indicates that “fat” and “sheep meat” were equally selected as the top flavor descriptors for wet-aged patties. “Sheep meat” was the most selected aroma for both the wet- and dry-aged top-of-mind odor question, indicating this was the most intense odor attribute for both ageing methods. The top-of-mind odor term “meat” and flavor term “strong” had no equivalent in the CATA terms; strong, however, does not describe a flavor but rather an intensity of flavor, and in this study may indicate the wet-aged patty was more intense overall for flavor compared to dry-aged. “Meat” is a non-specific term that we do not consider a useful characterizing attribute for inclusion in future studies.

The dry- and wet-aged mutton volatile profile results are similar to recent studies comparing the volatile profiles of wet- and dry-aged beef with a general trend of increased concentrations of aldehydes, alcohols, ketones and pyrazines in the dry-aged profile relative to the wet-aged profile [21,51].

Cooked sheep meat flavor results from a complex culmination of processes such as lipid degradation, proteolysis, Strecker degradation, thiamine degradation and the Maillard reaction, which produce a variety of alcohols, aldehydes, ketones, pyrazines, pyrroles, furans, furfurals and thiazoles, and each can contribute in varying degrees to the final flavor/aroma of sheep meat [42,52]. The generation of these compounds can be influenced by cooking time and temperature [53], pH of the meat [23], meat moisture content [12] and meat ageing conditions [54]. In this study, we found no significant difference in the TM% for wet- and dry-aged patties in either the raw or cooked state. While it was expected that the dry-aged patties would have lower TM than wet-aged, this result is not unusual, as it is understood that much of the moisture loss during dry-ageing is from the surface/trim of the primal cut and the internal meat is relatively protected [55]. Ha, McGilchrist, Polkinghorne, Huynh, Galletly, Kobayashi, Nishimura, Bonney, Kelman and Warner [21] also found no differences in the TM of wet- and dry-aged beef.

The higher aldehyde concentration in the dry-aged samples suggests that more lipid oxidation has occurred than in the wet-aged samples, which is logical given that dry-aged meat does not have the same packaging protection afforded to wet-aged meat [56]. The FAME analysis supports this finding, with the higher concentration of the monounsaturated fatty acid cis-9-oleic acid methyl ester (the most abundant unsaturated FAME in our samples) found in the wet-aged patty compared to the dry-aged patty, indicating increased oxidation rates in the dry-aged patties [57]. These differences may be due to the increased exposure of dry-aged meat to oxygen in the atmosphere and or the extended cooking time required for the dry-aged patties, which is discussed further below.

Pyrazines predominantly arise during the Maillard reaction [58] and they are responsible for roasted, toasted, fried and cooked meat aroma/flavors [42,59]. It is logical that the dry-aged patties had higher levels of these compounds. Firstly, we found the dry-aged patties took longer to reach an internal temperature of 65 °C than the wet-aged patties (3 min 50 s vs. 3 min 5 s, respectively) and, therefore, they exhibited more surface browning. Secondly, the dry-aged patties had a higher pH than the wet-aged (Table 4) and Madruga and Mottram [23] have previously demonstrated that pyrazine formation is favored by increasing pH. Given there was no difference in the TM% between the raw dry- and wet-aged patties, it is proposed that the increased cooking time may be related to differences in thermal conductivity related to the proportion of bound water in the wet- and dry-aged patties; however, this cannot be confirmed.

Caramel flavors in sheep meat have been associated with 2,3 butanedione, and 2,3 pentanedione [30]; unfortunately, the chromatographic system used for the volatile analysis was unable to resolve these compounds and we cannot confirm if they were present at higher levels in the dry-aged sample. Metallic flavors have been associated with nonanal, decanal 2,4 (E,E) heptadienal, 2 ethyl furan [30] and 1-octen-3-one [60]. We did not quantify 2,4 (E,E) heptadienal, but we found twice as much 2 ethyl furan in the dry-aged sample compared to the wet-aged sample, more than twice the concentration of nonanal in the dry-aged sample compared to the wet-aged sample, approximately 5 times the concentration of decanal in the dry-aged sample compared to the wet-aged, but three times more 1-octen-3-one in the wet-aged sample compared to the dry-aged. While at first glance, these results may seem to indicate that the dry-aged sample is likely to have a more metallic flavor, it should be noted that the odor impact of 1-octen-3-one is very high and it has an odor threshold an order of magnitude higher than the other compounds (ppt vs. ppb). Therefore, it is proposed that this compound is contributing to the metallic flavor associated with the wet-aged patties [30,42].

In addition to the compounds discussed above, sheep meat has a number of unique species-specific volatile compounds that can influence its flavor and aroma and impact the consumer acceptance of sheep meat [42,61]. The effect of dry-ageing on these compounds has not been previously described. In the present study, a “sheepy” flavor was more associated with the wet-aged patties than dry-aged and the increased “sheepy” flavor in wet-aged patties could be attributed to two compounds: hexanoic acid was present at higher levels in the wet-aged samples and is associated with a “goaty” aroma [38], and skatole (3-methylindole) was also found in the wet-aged treatment, but not the dry-aged, and is associated with “barnyard”, “fecal” and “animal” odor [42,62]. Para-cresol (p-cresol), associated with a “stable” and “animal” odor [61], was found at very low concentrations in only the dry-aged samples and does not appear to have influenced consumer characterization of the dry-aged mutton flavor.

Other potentially “problematic” compounds did not appear to be affected by ageing method; the concentration of the branched chain fatty acids 4-methyloctanoic (MOA), 4-ethyloctanoic (EOA) and 4-methylnonanoic (MNA), which are implicated in ”mutton” flavor and consumer acceptance [62,63], were not influenced by ageing method, suggesting that dry-ageing has no impact on the background level of these compounds.

## 5. Conclusions

The CATA methodology employed in this study demonstrated that consumers could detect a difference in the aroma/flavor profile between wet- and dry-aged mutton. Dry-aged mutton was associated with increased “roasted” and “caramel” flavor notes and wet-aged mutton was associated with increased “metallic” and “sheepy” notes. The mutton flavor lexicon developed in this study is suitable for further investigations into the flavor attributes driving consumer liking (or disliking) of mutton.

Volatile profiling supported the consumer characterization of mutton flavor with increased levels of pyrazines, which provide roasted, toasted, fried and cooked meat flavors in the dry-aged mutton patties compared to the wet-aged. For the wet-aged patties, hexanoic acid and skatole, which both contribute “goaty”, “fecal”, “animal” and “barnyard” aromas, were found to be higher in the wet-aged patties compared to the dry-aged patties. The concentration of the branched chain fatty acids MOA, EOA and MNA were not influenced by ageing method.

## Figures and Tables

**Table 1:** CATA (check-all-that-apply) terms and source.

CATA Term	Sensorial Attribute Association	Source
Barnyard	odor	[30,31,32,33]
Livery	odor, flavor	[30,31,32,34]
Sheepy	odor, flavor	[30,31,32,33,35]
Juicy	texture	[30,32,34]
Sweet	odor, flavor	[30,31,32,35,36,37,38,39]
Earthy	odor, flavor	In-house descriptor
Metallic	odor, flavor	[31,32,34]
Savory	odor, flavor	In-house descriptor
Roasted	odor, flavor	[34,37,40,41,42]
Acidic	odor, flavor	[30,31,32]
Fatty	odor, flavor	[36,37,38,39,40,41]
Fishy	odor, flavor	[31,32,40,42]
Caramel	odor	[37,40,42]
Dairy	Odor, flavor	[33,34,36,38,39,41]
Sour	flavor	[30,31,32]
Green/Grassy	odor, flavor	[32,33,36,37,38,39,41]

**Table 2:** Effect of ageing method (dry vs. wet) on frequency (count) of top three descriptor terms for odor and flavor of cooked mutton patties for top-of-mind questions ^1^.

Descriptors	Frequency Count	
	Wet-Aged	Dry-Aged
**Odor responses**		
Sheep meat	25	17
Cooked	6	11
Meat	12	7
**Flavor responses**		
Cooked	- ^2^	10
Fat	11	7
Sheep meat	11	5
Strong	9	-

^1^ The questions posed were Question 1: “In terms of odor, what is top-of-mind when you first smell the sample?” and Question 2: “In terms of flavor, what is top-of-mind when you first taste the sample?”. ^2^ Term was not in the top three most selected terms for the specified treatment.

**Table 3:** Effect of ageing method (dry vs. wet) on frequency of selection (count) of check-all-that-apply (CATA) descriptor terms for cooked mutton patties and statistical significance (*p*-value).

CATA Term	Frequency Count		*p*-Value
	Wet-Aged	Dry-Aged	
Barnyard	10	4	0.058
Livery	11	9	ns ^1^
Sheepy	45	34	0.041
Juicy	38	39	ns
Sweet	9	13	ns
Earthy	27	23	ns
Metallic	4	0	0.046
Savory	18	26	ns
Roasted	19	32	0.006
Acidic	3	1	ns
Fatty	38	31	ns
Fishy	2	1	ns
Caramel	0	6	0.014
Dairy	2	3	ns
Sour	6	1	0.059
Green/Grassy	9	8	ns

^1^ ns = not significant, *p* value is >0.10.

**Table 4:** Effect of ageing method (dry vs. wet), standard deviation (S.D.), t-value (t) with subscript degrees of freedom and statistical significance (*p*-value) on total moisture and pH of mutton patties.

Parameter	Wet-Aged		Dry-Aged		t_4_	*p*-Value
	Mean	S.D.	Mean	S.D.		
pH raw patty	6.46	0.046	6.56	0.023	−3.13	0.035
moisture raw patty (%)	65.4	1.50	63.4	1.36	−1.69	ns ^1^
moisture cooked patty (%)	61.0	2.51	59.8	0.786	−1.190	ns

^1^ ns = not significant, *p* value is >0.10.

**Table 5:** Effect of ageing method (dry vs. wet) on fatty acid methyl ester concentration (mg/g) in raw mutton patties, standard deviation (s.d.), t-value (t) with subscript degrees of freedom and statistical significance (*p*-value).

Fatty Acid Methyl Ester mg/g Meat	Wet-Aged		Dry-Aged		t_2_	*p*-Value
	Mean	S.D.	Mean	S.D.		
Methyl octanoate (C8:0)	nd ^1^		nd			
Methyl decanoate (C10:0)	0.263	0.000	0.370	0.004	36.7	0.001
Methyl laurate (C12:0)	0.144	0.000	0.365	0.004	75.0	<0.001
Methyl tetradecanoate (C14:0)	2.30	0.003	4.73	0.089	38.4	0.001
Methyl palmitate (C16:0)	17.4	0.023	30.9	0.588	32.4	0.001
Methyl palmitoleate (C16:1)	1.58	0.003	3.92	0.071	46.7	<0.001
Methyl octadecanoate (C18:0)	16.6	0.020	4.92	0.167	−98.7	<0.001
Cis-9-oleic acid methyl ester (C18:1)	35.8	0.045	16.4	0.241	−112	<0.001
Methyl linoleate (C18:2)	1.14	0.009	5.04	0.094	58.1	<0.001
Methyl linolenate (C18:3)	0.127	0.000	0.129	0.001	4.45	0.047
Methyl arachidate (C20:0)	1.31	0.002	2.14	0.038	30.8	0.001
Methyl docosanoate (C22:0)	0.446	0.002	0.441	0.005	−1.21	ns ^2^
Methyl erucate (C22:1)	nd		nd			
Methyl lignocerate (C24:0)	nd		nd			

^1^ nd = not detected. ^2^ ns = not significant, *p* value is >0.10.

**Table 6:** Effect of ageing method (dry vs. wet) on volatile concentrations (ng/g) ^1^ for cooked mutton patties; standard deviation (S.D.), t-value (t) with subscript degrees of freedom, level of significance (*p*-value), the experimental Kovats retention indices (LRI), the identification method (ID) and quantification ion (QI) are shown.

Volatile	LRI	ID ^2^	QI	Wet-Aged		Dry-Aged		t_6_	*p*-Value
				Mean ^2^	S.D.	Mean ^2^	S.D.		
Aldehydes									
3−Metylbutanal	650	a	44	205,255	24,542	230,562	26,176	−1.41	ns ^1^
Pentanal	699	a	44	707,052	142,368	1,223,792	234,593	−3.77	0.009
Hexanal	799	a	56	922,702	98,013	3,008,952	358,034	−11.2	<0.001
Heptanal	899	a	70	462,093	36,295	1,396,726	190,730	−9.63	<0.001
(Z)−4−Heptenal	899	a	41	308,628	29,982	883,366	101,196	−10.9	<0.001
(E)−2−heptenal	959	b	83	7046	704	4455	338	6.63	0.001
Octanal	1003	a	43	123,278	9583	488,892	694,64	−10.4	<0.001
(E)−2−Octenal	1053	b	55	1540	200	1674	284	−0.77	ns
Nonanal	1100	a	57	327,109	37,093	884,041	151,502	−7.14	<0.001
(E)−2−Nonenal	1158	b	70	2358	346	4984	341	−10.82	<0.001
(E,Z)−2,6−Nonadienal/2,6−Nonadienal, (E,E)−	1158	b	41	1376	287	3299	598	−5.80	0.001
Decanal	1206	a	57	2539	248	10,397	1699	−9.15	<0.001
(E)−2−Decenal	1262	a	81	nd ^3^		1163	247	−9.01	<0.001
Undecanal	1319	b	142	nd		24,501	3963	−12.3	<0.001
(E,E)−2,4−Decadienal	1320	b	67	149	107	1494	308	−8.25	<0.001
Tridecanal	1510	a	67	nd		109	64	−1.98	0.095
Total Aldehydes				3,068,676	350,801	8,170,998	1,122,398	−8.68	<0.001
Alcohols									
1−Hexanol	869	a	43	45,159	16,798	112,736	21,868	−4.90	0.003
1−Heptanol	971	a	70	27,582	2913	89,675	18,058	−6.79	<0.001
1−octen−3−ol	986	a	69	nd		3533	672	−10.4	<0.001
2−Ethyl−1−hexanol	1029	b	57	13,877	7552	5657	641	2.17	0.073
1−Octanol	1069	a	56	14,306	1119	49,350	10,715	−6.51	0.001
Total Alcohols				100,974	18,603	260,951	51,217	−5.87	0.001
Alkanes									
Octane	800	a	114	67,221	4529	59,945	6922	1.76	ns
Nonane	900	a	57	223,994	50,165	740,670	101,231	−9.15	<0.001
Decane	1000	a	57	10,014	5197	33,965	14,424	−3.12	0.02
Unadecane	1100	a	57	325,273	37,971	882,784	150,946	−7.16	<0.001
Dodecane	1200	a	57	4472	643	8572	1065	−6.59	0.001
Tridecane	1300	a	57	2801	518	3962	772	−2.50	0.047
Tetradecane	1400	a	57	4264	1962	6791	908	−2.34	0.058
2,6,10−Trimethyltridecane	1472	a	57	4657	685	4464	605	0.42	ns
Pentadecane	1500	a	57	1411	360	1920	567	−1.52	ns
Total Alkanes				644,106	91,439	1,743,072	259,186	8.00	<0.001
Aromatic hydrocarbons									
Toluene	762	b	91	90,840	7380	100,855	16,137	−1.13	ns
1,4−Dimethylbenzene (p−xylene)	864	a	91	42,008	2825	30,306	5428	3.83	0.009
Benzaldehyde	957	a	106	90,489	7344	93,882	15,105	−0.40	ns
Benzyl alcohol	1032	b	108	4575	1194	4407	340	0.27	ns
Benzeneacetaldehyde	1041	a	91	37,108	4825	73,883	14,767	−4.73	0.003
Acetophenone	1062	b	105	1848	233	1113	161	5.20	0.002
p−cresol	1076	b	107	nd		909	263	−4.55	0.004
Total Aromatic hydrocarbons				267,068	21,976	305,354	51,200	−1.37	0.218
Furans									
2−Ethylfuran	707	b	81	20,001	1395	36,727	2689	−11.04	<0.001
2−Furfural	824	b	96	117,820	7671	140,972	25,466	−1.74	ns
2−Pentylfuran	992	b	81	13,244	1639	47,256	6183	−10.6	<0.001
4−Hydroxy−2,5−dimethyl−3(H)furanone	1057	b	43	1792	323	1751	172	0.23	ns
Total Furans				152,858	9421	226,706	30,597	−4.61	0.004
Ketones									
2−Heptanone	889	a	58	25,585	2067	60,185	1222	−5.58	0.001
1−Octen−3−one	986	b	55	72,185	16,335	23,310	12,972	4.69	0.003
2−Nonanone	1089	a	58	6857	778	31,597	7269	−6.77	0.001
Total ketones				104,627	18,338	115,092	14,746	−0.89	0.408
Lactones.									
γ−Octalactone	1259	a	85	1131	220	8862	1709	−8.97	<0.001
γ−Nonalactone	1363	b	85	680	188	4359	808	−8.86	<0.001
Total Lactones				1811	364	13,221	2498	−9.04	0.002
Indoles									
indole	1292	b	117	nd		nd			
Skatole	1366	b	130	64,579	23,761	nd		5.42	0.002
Organic acids and their derivatives									
Butanoic acid	782	a	60	127,546	13,747	120,296	14,986	0.71	ns
Ethyl−butanoate	800	b	71	326,918	33,243	542,699	57,013	−6.54	0.001
Hexanoic acid	983	a	60	782,242	66,752	505,567	112,317	4.24	0.005
Octanoic acid	1173	a	60	23,446	4832	14,409	3355	3.07	0.022
4−methyl octanoic acid	1236	b	57	nd		nd			
Nonanoic acid	1268	b	60	1931	518	1360	500	1.59	ns
4−methyl nonanoic acid	1327	b	57	1659	411	1372	270	1.17	ns
4−ethyl octanoic acid	1333	b	57	4446	741	6306	1813	−1.90	ns
Total Organic acids				1,268,188	99,683	1,192,010	181,986	0.73	0.490
Pyrazines and Pyrroles									
Methyl−pyrazine	824	b	94	12,031	1848	15,412	3005	−1.92	ns
2,6−Dimethyl pyrazine/2,5−Dimethyl pyrazine	907	b	108	14,783	1178	25,568	3222	−6.29	0.001
Trimethyl pyrazine	1003	b	42	54,011	3661	215,486	33,875	−9.48	<0.001
2−Ethyl−3,6−Dimethyl−pyrazine/2−Ethyl−3,5−Dimethyl pyrazine	1076	a	135	3552	671	7613	1420		0.002
2,3−diethyl−5−methyl−Pyrazine	1159	b	150	200	71	338	74	−2.70	0.036
2−Acetylpyrrole	1058	b	94	nd		2193	176	23.8	<0.001
Total Pyrazines				86,769	6640	264,518	41,216	−8.52	0.003
Sulphur−containing compounds									
Dimethyl disulphide	741	b	94	6319	570	2699	1045	6.08	0.001
2−Acetylthiazole	1016	b	127	1123	303	1748	433	−2.37	0.056
2−Acetyl−2−thiazoline	1100	b	43	173,585	20,231	457,200	79,277	−6.93	<0.001
Total sulphur compounds				181,028	20,862	462,298	80,010	−6.80	<0.001
Terpenes									
α−Terpineol	1189	a	59	5265	697	7521	1021	−3.65	0.011
Neophytadiene	1838	b	95	1816	266	1692	271	0.66	ns
Total Terpenes				7081	943	9213	1193	−2.80	0.031
Total Volatiles				5,945,954	601,235	12,750,414	1,820,429	−7.65	<0.001

^1^ Determined semi-quantitatively using an assumed response factor of 1 peak area unit = 1 ng of compound; results are expressed as ng/g of meat. ^2^ Identification method; a = LRI match with published data and spectral match with NIST library or external standard, b = LRI match with published data and detection of quantification ion. ^3^ nd = not detected.

## Data Availability

The data used to support the findings of this study can be made available by the corresponding author upon request.
